# Prevalence of IgG4-Related Disease in Japan Based on Nationwide Survey in 2009

**DOI:** 10.1155/2012/358371

**Published:** 2012-07-31

**Authors:** Kazushige Uchida, Atsushi Masamune, Tooru Shimosegawa, Kazuichi Okazaki

**Affiliations:** ^1^Division of Gastroenterology and Hepatology, The Third Department of Internal Medicine, Kansai Medical University, 2-3-1 Shinmachi, Hirakata, Osaka 573-1197, Japan; ^2^Department of Gastroenterology, Tohoku University Graduate School of Medicine, Seiryo-Cho, Obaku, Sendai 980-8514, Japan

## Abstract

The number of patients with autoimmune pancreatitis who visited hospitals in Japan in 2007 was approximately 2709 (95% confidence interval; range 2540–3040). Because IgG4-related disease is a new clinical entity, there are no data with regard to its prevalence. To estimate the number of patients with IgG4-related disease in Japan, we randomly selected hospitals using stratification and asked them how many patients they had with IgG4-related disease in 2009. The number of patients with Mikulicz's disease, IgG4-related retroperitoneal fibrosis, IgG4-related renal disease, IgG4-related pulmonary disease, and IgG4-related lymphadenopathy who visited hospitals in Japan in 2009 was approximately 4304 (95% confidence interval; range 3360–5048), 272 (95% confidence interval; range 264–306), 57 (95% confidence interval; range 47–66), 354 (95% confidence interval; range 283–424), and 203 (95% confidence interval; range 187–240), respectively. The total number of patients with IgG4-related disease without autoimmune pancreatitis in Japan was approximately 5190 (95% confidence interval; range 4141–6084). The male : female ratio was 1 : 0.77, and the average of age of disease onset was 58.8 years. The total number of patients with IgG4-related disease in Japan in 2009, including autoimmune pancreatitis, was approximately 8000.

## 1. Introduction

IgG4-related disease (IgG4-RD) has recently been proposed as a new disease entity, and a number of case reports and studies evaluating the clinical characteristics of IgG4-RD have appeared in the literature. In 1995, Yoshida et al. proposed autoimmune pancreatitis (AIP) [1]. Hamano et al. reported that these patients showed elevated serum IgG4 [2]. Recently, autoimmune pancreatitis has been distinguished variously as type 1 and type 2 [3]. Type 1 AIP is characterized by IgG4. On the other hand, type 2 AIP is characterized by neutrophil infiltration. Type 1 AIP is commonly complicated with other organ involvement (OOI) [4, 5]. Kamisawa et al. proposed IgG4-related sclerosing disease [6]. This concept is based on sclerosing fibrosis. Systemic IgG4-related plasmacytic syndrome (SIPS) and IgG4-positive multiorgan lymphoproliferative syndrome (IgG4-MOLPS) were proposed based on lymphoproliferation [7, 8]. The Research Program for Intractable Disease by the Ministry of Health, Labor and Welfare (MHLW) has agreed to use the term “IgG4-related disease (IgG4-RD)” [9]. The most common OOIs are the well-known Mikulicz's disease, IgG4-related retroperitoneal fibrosis, IgG4-related renal disease, IgG4-related pulmonary disease, and IgG4-related lymphadenopathy. However, there has been no epidemiological report regarding the prevalence of IgG4-RD, even in a restricted area. We conducted a national survey for IgG4-RD, based on a national survey for AIP in 2009.

## 2. Methods

In 2006, the Japan Pancreas Society first proposed the diagnostic criteria for AIP [10, 11]. In 2007, using these criteria, a second nationwide survey for AIP was conducted and estimated the prevalence of AIP in Japan [12]. Briefly, following the guidelines of the Nationwide Epidemiological Survey Manual issued by the Research Committee on the Epidemiology of Intractable Diseases, [13] hospitals using stratification were randomly selected according to the number of beds in each; the more beds a hospital had, the greater the probability that it would be selected. Furthermore, 37 departments in hospitals in a particular stratum which were considered to have recorded AIP cases for research purposes and 317 departments in university hospitals were stratified separately. Next, the following departments were selected: Internal Medicine, Gastroenterology, Surgery, and Gastroenterological Surgery. From these selections, 2972 departments were nominated. A questionnaire was sent to the selected departments along with the diagnostic criteria for AIP, and respondents were asked to provide the number and sex of AIP patients who had visited the hospital in 2007. This survey included questions about sex, age of disease onset, and the diagnostic basis for AIP. According to this national survey of AIP, the number of AIP patients who visited a hospital in Japan in 2007 was estimated to be 2790 (95% confidence interval; range 2540–3040). AIP in the Japanese population was estimated as 0.82 per 100,000. The number of newly contracted patients in one year was 1,120 patients (95% confidence interval; range 1,000–1,240). Japanese vital statics of the Ministry of Health, Labor and Welfare for 2007 showed the total population to be 127,771,000 people (adult population, 104,197,000). 36.05% of the autoimmune pancreatitis patients were in the 250 hospitals who responded to the autoimmune pancreatitis nationwide survey in Japan. It was assumed that 2.773 times the number of presumptive patients in these 250 hospitals would be the number of estimated patients for the whole country. There were many experiences of autoimmune pancreatitis in these 250 hospitals. It seemed that patients with IgG4-related disease gathered a lot in these 250 hospitals. A questionnaire was sent to selected departments (Respiratory Medicine, Rheumatology, Internal Medicine (except Gastroenterology), Otolaryngology, Ophthalmology, and Urology) in these 250 hospitals, and respondents were asked to provide the number of IgG4-RD patients who had visited the hospital in 2009. This survey included questions about sex, age of disease onset, and the diagnostic basis for IgG4-RD.

## 3. Results

**Figure 1:**
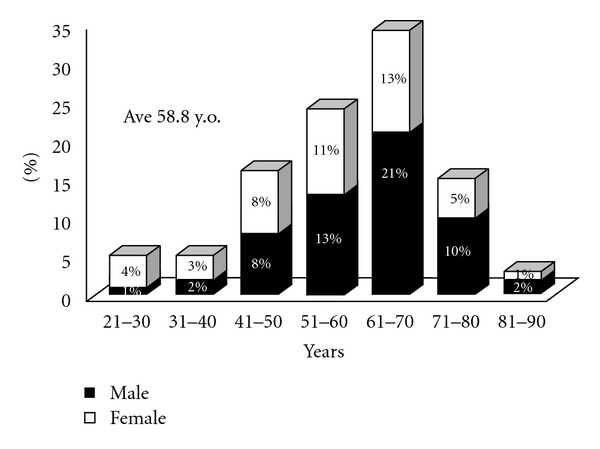
Sex and age of onset of IgG4-related disease. The male : female ratio was 1** **: 0.77, and the average of age of disease onset was 58.8 years.

In these patients, the male :  female ratio was 1 : 0.77. Figure 1 shows the distribution of the age of these patients at disease onset. The average of age of disease onset was 58.8 years. The peak was in the age range 61–70 years, and the disease-onset age in approximately one-third of the patients (33%) was 61–70 years. Interestingly, the number of patients with a disease-onset age of less than 40 years was dramatically lower, as most of the patients (90%) started to show IgG4-RD after the age of 40.

**Figure 2:**
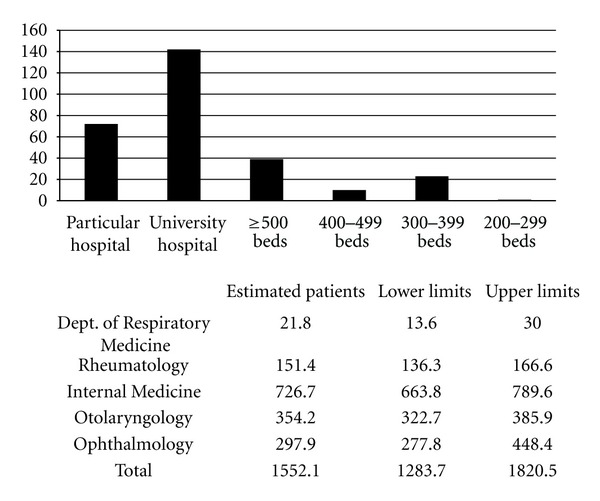
Mikulicz' disease without autoimmune pancreatitis. The number of patients with Mikulicz's disease without autoimmune pancreatitis, who visited hospitals in Japan in 2009, was approximately 4304 (95% confidence interval; range 3360–5048).

**Figure 3:**
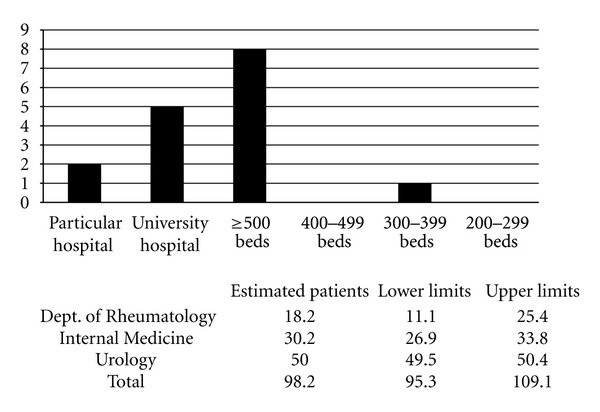
IgG4-related retroperitoneal fibrosis without autoimmune pancreatitis. The number of patients with IgG4-related retroperitoneal fibrosis without autoimmune pancreatitis, who visited hospitals in Japan in 2009 was approximately 272 (95% confidence interval; range 246–303).

**Figure 4:**
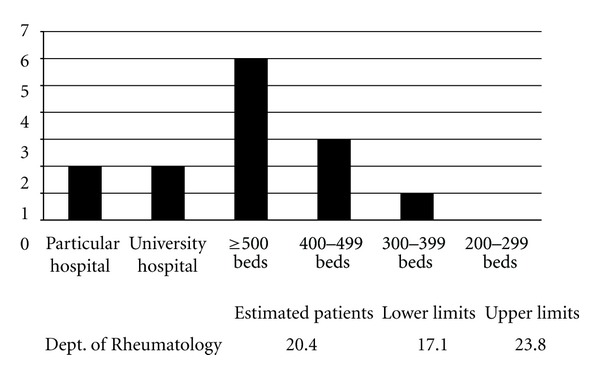
IgG4-related renal disease without autoimmune pancreatitis. The number of patients with IgG4-related renal disease without autoimmune pancreatitis, who visited hospitals in Japan in 2009, was approximately 57 (95% confidence interval; range 47–66).

**Figure 5:**
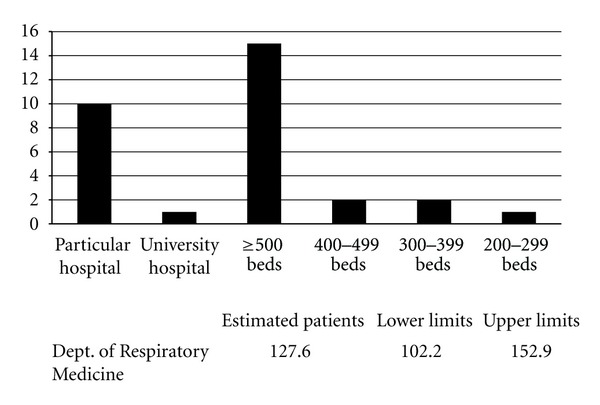
IgG4-related pulmonary disease without autoimmune pancreatitis. The number of patients with IgG4-related pulmonary disease without autoimmune pancreatitis, who visited hospitals in Japan in 2009, was approximately 354 (95% confidence interval; range 283–424).

**Figure 6:**
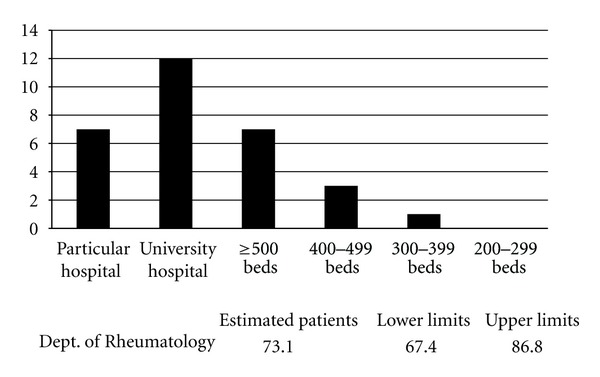
IgG4-related lymphadenopathy without autoimmune pancreatitis. The number of patients with IgG4-related lymphadenopathy without autoimmune pancreatitis, who visited hospitals in Japan in 2009, was approximately 203 (95% confidence interval; range 187–240).

A total of 301 (26.8%) of 1250 departments responded to the questionnaire (Table 1). Based on these results, the number of patients with Mikulicz's disease without autoimmune pancreatitis who visited hospitals in Japan in 2009, was approximately 4304 (95% confidence interval; range 3360–5048) (Figure 2). The number of patients with IgG4-related retroperitoneal fibrosis without autoimmune pancreatitis who visited hospitals was approximately 272 (95% confidence interval; range 246–303) (Figure 3). The number of patients with IgG4-related renal disease without autoimmune pancreatitis, who visited hospitals was approximately 57 (95% confidence interval; range 47–66) (Figure 4). The number of patients with IgG4-related pulmonary disease without autoimmune pancreatitis who visited hospitals was approximately 354 (95% confidence interval; range 283–424) (Figure 5). The number of patients with IgG4-related lymphadenopathy without autoimmune pancreatitis who visited hospitals was approximately 203 (95% confidence interval; range 187–240) (Figure 6). The total number of patients with IgG4-related disease without autoimmune pancreatitis in Japan was estimated to be approximately 5190 (95% confidence interval; range 4141–6084).

## 4. Discussion

This is one of several nationwide surveys conducted to elucidate the number of AIP patients in Japan and also the first such survey to be conducted worldwide. It is difficult to ascertain the number of patients with IgG4-RD, the awareness of this disease is low, and its symptoms are varied. Another national survey in Japan was reported by Umehara et al. [9]. They have estimated the number of individuals with IgG4-RD throughout Japan by using the number of patients in Ishikawa Prefecture as an example. Populations in Ishikawa Prefecture contains 1.16 million people, with little population inflow/outflow. In Ishikawa Prefecture, there are two University Hospitals, Kanazawa Medical University Hospital and Kanazawa University Hospital. Assuming that new patients with IgG4-RD would visit one of the two university hospitals, it was estimated that the incidence of this disease throughout Japan is 0.28–1.08/100,000 population with 336–1,300 patients newly diagnosed per year from 2003 to 2009. Since the median age of onset of IgG4-RD is 58 years and the clinical symptoms are relatively mild, with slow progression and good response to steroid therapy, life expectancy after diagnosis has been estimated at 20 years. Thus, it has been estimated that there are approximately 6,700 to 26,000 patients in Japan who have developed IgG4-RD over the past 20 years. From our national survey, the total number of patients with IgG4-RD without autoimmune pancreatitis in Japan was approximately 5190 (95% confidence interval; range 4141–6084). The number of AIP patients who visited a hospital in Japan was estimated to be 2790 patients. Therefore, the total number of patients with IgG4-related disease including autoimmune pancreatitis in Japan in 2009 was approximately 8000. Our estimate is somewhat lower than another national survey from Umehara et al. There are several possibilities of reasons for this matter. In this survey, we estimated the number of IgG4-RD patients based on the hospitals' treatment of AIP. On the other hand, Umehara et al.'s result was estimated from the two university hospitals (Department of Rheumatology) in Ishikawa Prefecture. From these two national surveys, it is suggested that total number of patients with IgG4-related disease including autoimmune pancreatitis in Japan was approximately about 10,000 and the average of age of disease onset was 58 years.

It has been reported the ratio of male patients in autoimmune pancreatitis [5]. In this survey, the male : female ratio was 1 : 0.77. Most patients have Mikulicz's disease without autoimmune pancreatitis. In Mikulicz's disease, the male : female ratio was 1.30 : 1. This is the reason that the deference is few at male and female ratio compared with autoimmune pancreatitis. Answer rate of this survey is not so high. One reason is guessed that IgG4-RD is not familiarized, therefore the patients with IgG4-RD are concentrated on the hospital that answered this survey. In 2011, the comprehensive diagnostic criteria for IgG4-RD are established by all Japan G4 team [14]. It will be necessary to familiarize general physicians with this new disease concept. 

## Figures and Tables

**Table 1:** Stratification and selection of hospitals and survey results.

Stratification	Hospitals nominated	Department nominated	Departments replying	Reply rate (%)
University hospital	49	245	58	23.7
Particular hospital^a^	55	275	96	34.9
≥500 beds	72	360	99	27.5
400–499 beds	33	165	38	23.0
300–399 beds	27	135	33	23.0
200–299 beds	12	60	10	16.7
100–199 beds	1	5	0	0
≤99 beds	1	5	0	0

Total	250	1250	301	26.6

^a^Hospitals considered to have collected AIP cases for research purposes.
